# Antioxidant and Anti-Inflammatory Effects of Korean Black Ginseng Extract through ER Stress Pathway

**DOI:** 10.3390/antiox10010062

**Published:** 2021-01-06

**Authors:** Mi-Yeong An, So Rok Lee, Hye-Jeong Hwang, Ju-Gyeong Yoon, Hae-Jeung Lee, Jin Ah Cho

**Affiliations:** 1 Department of Food and Nutrition, Chungnam National University, 99, Daehak-ro, Yuseong-gu, Daejeon 34134, Korea; miyeong1119@cnu.ac.kr (M.-Y.A.); sj807sr@cnu.ac.kr (S.R.L.); jugyeongs2@gmail.com (J.-G.Y.); 2 Department of Agrofood Resources, National Institute of Agricultural Sciences, Rural Development Administration, Wanju 55365, Korea; hjh1027@korea.kr; 3 Department of Food and Nutrition, Gachon University, 1342 Seongnam-daero, Sujeong-gu, Seongnam-si, Gyeonggi-do 13120, Korea

**Keywords:** Korean black ginseng, anti-inflammatory, antioxidant, ER stress

## Abstract

The excessive release of reactive oxygen species (ROS) can result in the development of chronic inflammation. The mechanisms involved in inflammation are various, with endoplasmic reticulum (ER) stress known to be among them. We have previously shown that black ginseng (BG) reduced lipid accumulation in and enhanced the antioxidant function of the liver in vitro and in vivo mostly due to ginsenoside Rb1, Rg3 and Rk1 components. Therefore, this study investigated the antioxidant effect of BG on the intestines and its possible mechanistic pathway through ER stress. The results showed that BG extract decreased ROS and nitric oxide (NO) production and reduced inducible nitric oxide synthase (iNOS) expression levels in vitro, and these results were confirmed by zebrafish embryos in vivo. However, this phenotype was abolished in the absence of inositol-requiring enzyme 1 (IRE1α) but not in the absence of protein kinase RNA (PKR)-like ER-resistant kinase (PERK) or X-box-binding protein 1 (XBP1) in the mouse embryo fibroblast (MEF) knockout (KO) cells, suggesting that BG elicits an antioxidant effect in an IRE1α-dependent manner. Antioxidant and anti-inflammatory effects were assessed in the liver and intestines of the mouse model affected by nonalcoholic fatty liver disease (NAFLD), which was induced by a high-fat/high-fructose diet. In the liver, BG treatment rescued NAFLD-induced glutathione (GSH), catalase (CAT), tumor necrosis factor-α (TNF-α) and interleukin (IL)-6 expression. In the intestines, BG also rescued NAFLD-induced shortened villi, inflammatory immune cell infiltration, upregulated IL-6, cytosine-cytosine-adenosine-adenosine-thymidine (CCAAT)/enhancer-binding homologous protein (CHOP) and binding immunoglobulin protein (BiP) expression. In conclusion, our results show that BG reduces ROS and NO production followed by inflammation in an IRE1α-dependent and XBP1-independent manner. The results suggest that BG provides antioxidant and anti-inflammatory effects through an ER stress mechanism.

## 1. Introduction

Inflammation is the first immune response to infection, and the mechanism involved in inflammation is complex. When harmful substances such as bacteria or viruses enter the body, immune cells detect them and secrete various inflammatory mediators [1]. Nitric oxide (NO), a free radical inorganic signaling molecule, is involved in many physiological processes, including the regulation of blood pressure, immune response and neural communication. Excessive release of NO can cause the development of various diseases, including chronic inflammation. NO is synthesized from L-arginine by NO synthases (NOS), which are divided into constitutive NOS and inducible NOS (iNOS) [2]. In particular, it is reported that iNOS is expressed in various cells such as smooth muscle cells, hepatocytes, macrophages and monocytes following the stimulation of various inflammatory cytokines. Induction of iNOS can result in excessive tissue damage, genetic mutations, nerve damage, increased vascular permeability, and most of all, inflammation [3].

Reactive oxygen species (ROS), often induced by iNOS, are normal cellular metabolites produced during the oxidation response of mitochondrial respiration, playing an important role in activating signaling pathways in various intracellular and extracellular environmental conditions [4]. Although an appropriate amount of ROS has a positive effect on removing invading pathogens and recovering from wounds, excessive ROS interferes with oxidation–reduction homeostasis and affects a variety of diseases, including inflammatory-related metabolic syndromes, cardiovascular disease and cancer [5,6,7]. In particular, ROS in the intestines activates C-Jun N-terminal kinase (JNK), protein kinase C (PKC) and nuclear factor kappa light chain enhancer of activated B cells (NF-κB), resulting in damage to the intestinal barrier as well as disruption of microbial balance. In addition, ROS has been reported to activate NF-κB directly through other stimuli such as tumor necrosis factor-α (TNF-α), interleukin-1β (IL-1β) and IL-6 [8,9].

The endoplasmic reticulum (ER) has significant roles in the synthesis, folding and maturation of proteins. Failure of ER homeostasis leads to ER stress, unfolded protein response (UPR) and apoptosis [10,11]. Malnutrition, hypoxia, oxidized lipids and loss of calcium homeostasis can increase ROS production, which eventually induces ER stress [12]. ER stress activates unfolded protein response (UPR) signaling with its three sensors consisting of inositol-requiring enzyme 1 (IRE1), protein kinase RNA-like endoplasmic reticulum kinase (PERK) and activating transcription factor 6 (ATF6). Upon UPR activation, ER luminal chaperone binding immunoglobulin protein (BiP) binds to three ER stress sensors in the resting state, dissociates from the ER stress sensors and binds to unfolded proteins. Upon BiP release from ER stress sensors, IRE1, ATF6 and PERK undergo post-translational modifications and start the transcription and translation programs that define UPR. When ER stress increases, activated PERK signaling decreases protein synthesis through phosphorylation of eukaryotic initiation factor 2 α (eIF2α) and eventually promotes the expression of cytosine-cytosine-adenosine-adenosine-thymidine (CCAAT)/enhancer-binding homologous protein (CHOP), a major regulator of apoptosis. X-box-binding protein 1 (XBP1) is a downstream signal transduction molecule of IRE1, which processes full-length XBP1 (XBP1u) mRNA to produce a spliced form of the XBP1 protein (XBP1s). XBP1 encodes enzymes and genes used for the degradation of inappropriately folded proteins [13]. Recently, increased ER stress and ROS production were observed in several diseases, suggesting ROS as a crucial regulator of ER stress, although the molecular link between ROS, and components of the ER stress machinery are still obscure.

Ginseng has been used in Asian countries as a conventional herbal medicine that positively affects vasodilation and has anti-inflammatory, antioxidant and anticancer effects [14,15]. Raw ginseng can be processed to form red ginseng or white ginseng to enhance its efficacy and preservation properties. Black ginseng (BG) is a new style of processed ginseng manufactured from white ginseng with nine steaming cycles, which turns the ginseng black in color. It has been reported that a frequent heatingimproves antioxidant activity [16]. BG has been found to contain relatively lower polar ginsenosides compared to the white and red ginseng, and 19 ginsenosides were discovered such as Rb1, Rb2, Rc, Rd, Re, Rf, Rg1, Rg6, F4, Rk3, Rh4, 20(S)-, 20(R)-Rg3, 20(S)-, 20(R)-Rs3, Rk1, Rg5, Rs4 and Rs5 using high performance liquid chromatography-evaporative light scattering detector (HPLC-ELSD), Our previous study confirmed and identified composition and amounts of ginsenosides of BG, such as Rg1, Re, Rf, Rh1(S and R), Rh2 (S and R), Rb1, Rc, F1, Rb2, Rb3, F2, Rg3 (S and R), PPT (S and R), K, Rh2 (S and R), PPD and total ginsenosides in the leaf and roots. However, few detailed mechanisms associated with anti-inflammatory and antioxidant activities of BG have been described. 

In this study, we explored the effect of BG on the inflammation, oxidation and ER stress in genetically engineered mouse embryonic fibroblast (MEF) cells in vitro and in a nonalcoholic fatty liver disease (NAFLD) mouse model in vivo as a metabolic syndrome related to inflammation.

## 2. Materials and Methods

### 2.1. Reagents and Chemicals

Korean ginseng was purchased from the Korea Ginseng Agricultural Cooperative Association (Gyeonggi-do, Korea) and prepared using standard production processes. Washed ginseng leaves and roots were briefly put in a strainer, and the processes of steaming and drying were repeated 9 times while providing water at 65 °C to 95 °C, followed by grinding. Extraction was performed for 24 h with 80% methanol to create a mixture 10 times the original weight. The extract was filtered, concentrated in a rotating evaporator, and freeze dried. BG extract was dissolved in 100% DMSO and used at 50 mg/mL to use in the study.

The interleukin-1β (IL-1β, JW CreaGene Co., Seongnam, Korea), tumor necrosis factor-α (TNF-α, JW Creagene Co., Seongnam, Korea), interferon-γ (IFN-γ, R&D Systems Inc., Minneapolis, MN, USA) and lipopolysaccharides (LPS, InvivoGen, San Diego, CA, USA) were purchased as indicated. 

### 2.2. Cell Culture

The MEF cells were a generous gift from David Ron (University of Cambridge, Cambridge, UK). Human intestinal epithelial Caco-2 cells were purchased from the American Type Culture Collection (USA). Cells were cultured in Dulbecco’s modified Eagle’s medium (Gibco, USA) added with 1% penicillin–streptomycin (Sigma-Aldrich Co., Saint Louis, MO, USA) and 10% heat-inactivated fetal bovine serum (Gibco, Grand Island, NY, USA) at 37 °C under an atmosphere of 5% CO_2_.

### 2.3. Cell Viability

Cell viability was performed using the water-soluble tetrazolium (WST) assay kit (EZ Cytox, Daeil Lab Service Co. Ltd., Seoul, Korea) according to the manufacturer’s instructions. Wild-type (wt) MEF cells were seeded in 96-well cell culture plates (Hyundai Micro Co., Seoul, Korea) at 2.5 × 10^4^ cells/well, and Caco-2 cells were seeded in 96-well cell culture plates at 5 × 10^4^ cells/well. The cells were pretreated with BG (0, 1, 10, 50, 100, 250, 500 µg/mL) for 24 h or 48 h. Cytotoxicity was quantified after 4 h by measuring the absorbance at 450 nm with a microplate reader (xMark™ microplate absorbance spectrophotometer, Bio-Rad Inc., Hercules, CA, USA). The survival rate of cells at the indicated treatment concentration is expressed as a percentage (%) based on the survival rate of the untreated control group.

### 2.4. Zebrafish Husbandry and Embryos Collection

Zebrafish (Danio rerio) embryos were supplied by the Zebrafish Center for Disease Modeling (ZCDM, Korea) and maintained in a temperature-controlled room at 28 °C with a 14:10 h day/night cycle. Zebrafish were fed with brine shrimp 4 times per day. Experiments were performed following the Animal Research Guidelines at Chungnam National University (CNU-01027). At 0.75 to 50 h post fertilization (hpf), zebrafish embryos were isolated in 24-well cell culture plates (Hyundai Micro Co., Seoul, Korea) at 10 embryos/well with egg water supplemented with 0.1% methylene blue, then treated with BG (0, 1, 10, 50, 100, 250 µg/mL) for various times as indicated. Egg water supplemented with BG was changed every 24 h. Embryos were examined using an optical microscope (DM2000, Leica co., Wentzler, Germany), and zebrafish larvae were examined using a stereo microscope (SZ2-ILST, Olympus, Tokyo, Japan) to assess embryo viability. All experiments on the zebrafish were performed according to the protocol approved by the Animal Care and Use Committee of Chungnam National University (CNU-01027).

### 2.5. Animals and Experimental Diets

Four-week-old male ICR mice were purchased from Orient Bio Co. Ltd. (Gyeonggi-do, Korea) and housed in a light-controlled room (12:12 h day/night cycle) at 20–25 °C. After 1 week of quarantine, the mice were fed either a normal diet (Research diet, NJ, USA) with normal drinking water (NC) or a 45% high-fat diet (Research diet, NJ, USA) with 10% fructose in the drinking water (high-fat/high-fructose diet, HC). After NAFLD was induced, mice were randomly divided into 5 groups: normal diet (NC, *n* = 10), HC diet (HC, *n* = 9), HC with 0.5% BG (low dose of BG (LB), *n* = 9), HC with 1% BG (medium dose of BG (MB), *n* = 9) and HC with 2% BG (high dose of BG (HB), *n* = 9). The diets supplemented with BG (0.5%, 1%, 2%) were fed to mice for 8 weeks. The detailed experimental design is shown in Table 1. All animal experiments were approved by the Committee of Animal Care and Experimentation of Gachon University (GIAUAC-R2017019) and were carried out in accordance with the National Institutes of Health Guide for the Care and Use of Laboratory Animals (NIH Publications No. 8023, revised 1978).

### 2.6. NO Measurement

Nitric oxide (NO) assay was performed according to the manufacturer’s instructions using the Griess reagent system (Promega Co., Madison, WI, USA). MEF cell lines were seeded in 24-well cell culture plates at 5 × 10^4^ cells/well and pretreated with BG (0, 1, 10, 50, 100, 250 µg/mL) for 24 h, then subsequently a cytokine cocktail (CT: 50 ng/mL TNF-α + 50 ng/mL IFN-γ + 25 ng/mL IL-1β + 10 µg/mL LPS) stimulation for an additional 24 h. Absorbance was quantified at 540 nm on a microplate reader (xMark™ Microplate Absorbance Spectrophotometer, Bio-Rad Inc., Hercules, CA, USA).

### 2.7. ROS Measurement

MEF cell lines were seeded in 96-well cell culture plates (SPL Life Sciences Co., Pocheon, Korea) at 1.5 × 10^4^ cells/well and pretreated with BG (0, 1, 10, 50, 100, 250 µg/mL) for 23 h, then subsequently stimulated with 2 mM H_2_O_2_ for 1 h to induce ROS production. Then 10 μM 2′,7′-dichlorofluorescein diacetate (DCFH-DA, Sigma-Aldrich Co., Saint Louis, MO, USA) was added to the cells. After 1 h incubation at 37 °C in the dark, the florescence of DCFH-DA was measured at an excitation wavelength of 485 nm and an emission wavelength of 535 nm by using a fluorescence multimode detector (DTX800, Beckman Coulter, Inc., Brea, CA, USA).

For ROS measurement in vivo, zebrafish embryos at 8 hpf were isolated in 24-well plates at 10 embryos/well with egg water supplemented with 0.1% methylene blue, treated with BG (0, 1, 10, 50, 100, 250 µg/mL) for 1 h, and 5 mM H_2_O_2_ was added for an additional 24 h to induce ROS. After incubation, the embryos were washed with egg water and grown to 2-day post fertilization (dpf). At 2 dpf, the eggs were treated with egg water containing DCFH-DA (20 μg/mL), incubated for 1 h at 28 °C in the dark, and then washed with egg water. Images of stained embryos were observed using a digital microscope (Dino-Lite Digital Microscope, ANMO Electronics Co., New Taipei City, Taiwan). 

### 2.8. RNA Extraction, RT-PCR and Real-Time qPCR

The liver and intestine tissues of mice were homogenized using a gentleMACS™ Dissociator (Miltenyi Biotec Co., Bergisch Gladbach, Germany) with TRI reagent (MRC Inc., Cincinnati, OH, USA) for RNA extraction. Then, chloroform (Junsei Co., Tokyo, Japan) was added and the homogenate was centrifuged at 12,000× *g* for 15 min at 4 °C. The supernatant was collected, isopropanol (Duksan Co., Ansan-si, Korea) was added, and centrifuged at 12,000 × g for 8 min at 20 °C. The supernatant was removed, and the RNA concentration from the pellet was quantified using a NanoDrop spectrophotometer (Thermo Scientific Inc., Waltham, MA, USA). The cDNA was synthesized using the RT Kit (Biofact Co., Daejeon, Korea). In order to quantify the mRNA expression of binding immunoglobulin protein (BiP), CCAAT/enhancer-binding homologous protein (CHOP) and interleukin-6 (IL-6), reverse transcription polymerase chain reaction (RT-PCR) was performed using 2 × Taq Basic PCR Master Mix according to the manufacturer’s instructions (Biofact Co., Korea). The samples were loaded on to 2% agarose gel for electrophoresis and observed using a gel documentation system (AE-9000 E-Graph, ATTO Co., Tokyo, Japan) under UV light. For real-time quantitative PCR, cDNA was synthesized with SYBR Green Master Mix (Takara Bio, Otsu, Japan) and the mRNA expression was quantified using ABI QuantStudio 3 (Applied Biosystems, Foster City, CA, USA). Primers used in RT-PCR and the real-time qPCR experiments are shown in Table 2. 

### 2.9. Enzyme-Linked Immunosorbent Measurement (ELISA)

The liver tissues of mice were homogenized using a glass homogenizer with phosphate buffered saline (PBS) and ultrasonicated to further break the cell membranes. Then, homogenate was centrifuged at 1500× *g* for 15 min. The supernatant was collected to perform an ELISA assay for glutathione (GSH) and catalase (CAT) according to the manufacturer’s instructions (BlueGene Biotech, Shanghai, China). 

### 2.10. Western Blotting

MEF cells were pretreated with BG for 24 h followed by CT treatment for an additional 4 h. Cell lysate was extracted radio immunoprecipitation assay buffer (RIPA buffer, Thermo Scientific Inc., Illinois, USA) on ice and centrifuged at 20,000× *g* for 20 min at 4 °C. Protein concentration was measured using a bicinchoninic acid (BCA) protein assay kit (Thermo Scientific Inc., USA) and samples were loaded onto 10% sodium dodecyl sulfate (SDS) polyacrylamide gel for electrophoresis and then transferred to nitrocellulose membrane (Bio-Rad Inc., Hercules, CA, USA). After blocking for 1 h with 5% nonfat milk in tris-buffered saline with 0.1% Tween 20 (TBST) buffer at room temperature, anti-iNOS rabbit antibodies (1:1000 dilution; Cell Signaling Technology, Illinois, USA) or anti-glyceraldehyde-3-phosphate dehydrogenase (GAPDH) mouse antibodies (1:1000 dilution; Thermo Scientific Inc., Illinois, USA) were added to the membrane and maintained overnight at 4 °C. Then, the secondary antibodies (1:10,000 dilution; Thermo Scientific Inc., Middlesex County, MA, USA) were added to the membranes for 1 h at room temperature. Enhanced chemiluminescence (West Femto Maximum Sensitivity Substrate Kit, Thermo Scientific Inc., Illinois, USA) and the ChemiDoc system (AE-9100 EZ Capture, ATTO Co., Tokyo, Japan) were used for detection.

### 2.11. Tissue Histology

The small intestine and colon tissues of mice were fixed in a 10% formaldehyde solution, processed into paraffin blocks using standard methods and stained with hematoxylin and eosin (H&E). The tissue sections were stained with H&E (T&P Bio, Gyeonggi-do, Korea) and all stained tissues were taken with an optical microscope (CX31, Olympus, Tokyo, Japan).

### 2.12. Statistical Analysis

Data were analyzed using SPSS 24.0 (SPSS Inc., Chicago, IL, USA) software. All experiments were conducted in triplicates. Results are expressed as mean ± standard deviation. Statistical analysis of comparisons between two groups was performed using Student’s t-test, while one-way analysis of variance (ANOVA) with Duncan’s multiple range test was used in the analysis of two or more groups. Statistical significance was assessed at a *p* < 0.05.

## 3. Results

### 3.1. Cytotoxic Effect of BG In Vitro

**Figure 1:**
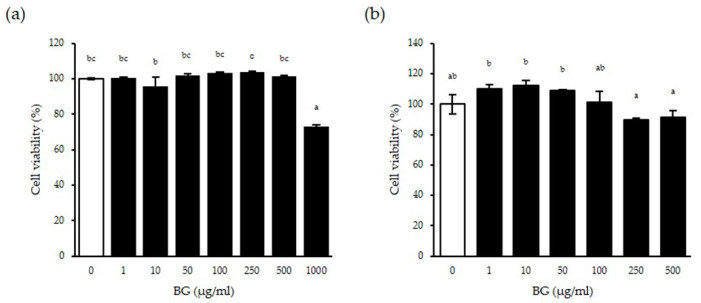
Effect of BG on cell viability in vitro. Cell viability was measured on wild-type mouse embryo fibroblast (wt MEF) for 24 h (**a**) and Caco-2 cells for 48 h (**b**) with the indicated concentration of BG. Cell viability of the negative control cells set as 100%. At least three independent experiments were performed. Data are expressed as means ± SD. Significant differences between different concentrations were analyzed using Duncan’s multiple range test with one-way ANOVA, and values labeled with different letters are significantly different (*p* < 0.05). White bar represents negative control. BG, black ginseng.

We analyzed the composition of black ginseng in the previously published paper and showed compositions and amount of ginsenosides in the leaf and roots of BG (Rg1, Re, Rf, Rh1 (S and R), Rh2 (S and R), Rb1, Rc, F1, Rb2, Rb3, F2, Rg3 (S and R), PPT (S and R), K, Rh2 (S and R), PPD and total ginsenosides) [16,17]. In this study, to evaluate the cytotoxicity of BG, WST analysis was performed on wt MEF cells and human intestinal epithelial Caco-2 cells. When the cells were treated with BG, cell viability decreased significantly at the 1000 µg/mL concentration of BG for 24 h on wt MEF cells (*p* < 0.001) (Figure 1a). Although there was significantly decreased viability from the 250 µg/mL concentration and the higher BG on Caco-2 cells for 48 h (*p* = 0.030), the cell viability was above 80% compared to the negative control, suggesting that there is no cytotoxicity of BG below 1000 µg/mL concentration on cells (Figure 1b). Based on these results, a maximum 250 µg/mL BG concentration was used in all subsequent in vitro experiments.

### 3.2. Inhibitory Effect of BG on Nitric Oxide (NO) Production

**Figure 2:**
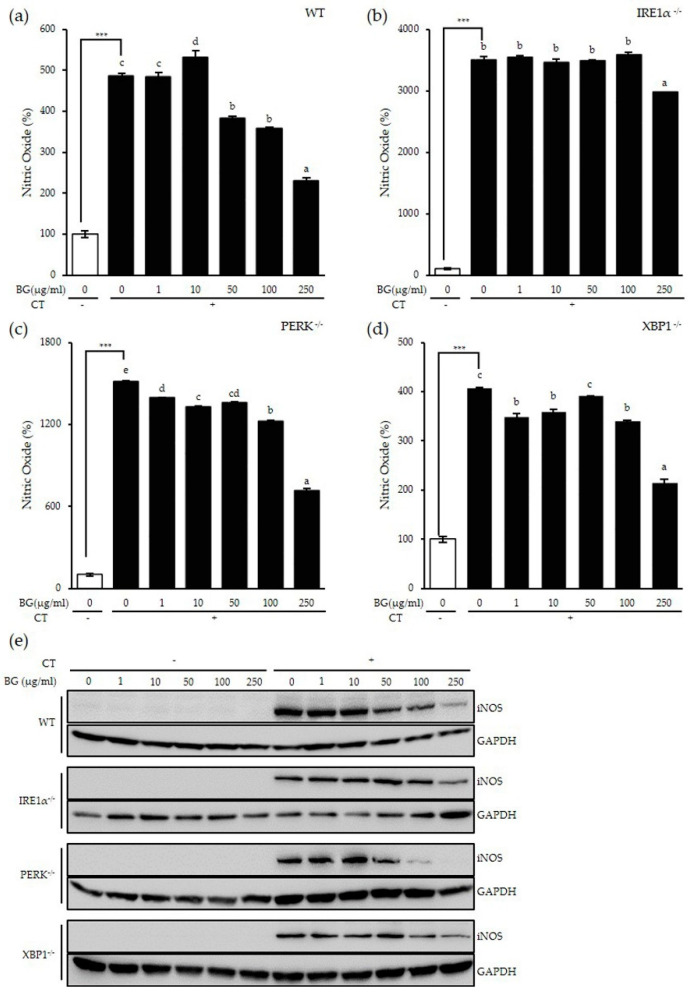
Inhibitory effect of BG on CT-induced nitric oxide (NO) production and induced NO synthases (iNOS) expression in an inositol-requiring enzyme 1 (IRE1α)-dependent way. The (**a**) wt, (**b**) IRE1α knockout (KO), (**c**) protein kinase RNA (PKR)-like endoplasmic reticulum -resistant kinase (PERK) KO and (**d**) X-box-binding protein 1 (XBP1) KO MEF cell lines were pretreated with various concentrations of BG for 24 h followed by CT (50 ng/mL TNF-α + 50 ng/mL interferon (IFN)-γ + 25 ng/mL IL-1β + lipopolysaccharides (LPS) 10 µg/mL) stimulation for an additional 24 h. Conditioned media were harvested to measure NO levels (**a**–**d**); cell lysates were extracted for Western blot (**e**). Glyceraldehyde-3-phosphate dehydrogenase (GAPDH) was the loading control. Data are shown as the means ± SD of three independent experiments. Significant differences between different concentrations were analyzed using Duncan’s multiple range test with one-way ANOVA and values labeled with different letters are significantly different (*p* < 0.05). Student’s t-test was performed to assess the difference between the control group and the CT-treated group in the absence of BG. *** *p* < 0.001. White bar represents negative control. BG, black ginseng; CT, cytokine cocktail.

Previously, we published HPLC analysis of BG showing the total ginsenoside contents and the compositions and the amount of ginsenosides in the BG roots and leaves [17,18]. There were 22 different types of ginsenoside with varied composition in BG, and the most abundant components were F2, Rg2 and Rg3. The total ginsenoside content was 22.94 mg/g in the leaves and 21.00 mg/g in the roots. We showed that these ginsenosides of BG reduced lipid accumulation in the liver, suggesting the anti-obesity effect of BG. Using this same extract, we investigated NO production in the presence of BG in our in vitro system. NO production was measured in MEF cells pretreated with various concentrations of BG for 24 h followed by an additional 24 h treatment with CT. NO production in the negative control was set to 100%, which increased significantly with CT treatment as expected (*p* < 0.001) (Figure 2). In wt MEF cells, cytokine-induced NO production was significantly reduced with BG treatment in a dose-dependent manner at concentrations of 50 µg/mL and higher, suggesting that BG exhibits an antioxidant effect starting at a concentration of 50 µg/mL (Figure 2a).

Next, MEF knockout (KO) cells, which lacked ER stress sensors, were used to investigate the mechanism associated with this antioxidant effect. IRE1α is a major ER stress sensor in addition to two other sensors (PERK and ATF6). Unlike wt MEF cells, cytokine-induced NO production was not affected by BG treatment in IRE1α KO MEF cells (Figure 2b). On the other hand, PERK KO MEF cells and XBP1 KO MEF cells showed significant reduction of NO production dose-dependently by BG treatment (Figure 2c,d). Although XBP1 is the downstream molecule of IRE1α but not necessarily, our data showed that XBP1 is not involved in this pathway. Therefore, these results show that BG has an anti-oxidative effect in the presence of inflammation in an IRE1α-dependent and XBP1-independent manner.

To confirm this result, iNOS catalysis of NO was investigated (Figure 2e). There was no iNOS expression in the absence of stimulation; however, CT stimulation significantly increased iNOS protein expression in all cell lines. The CT-induced iNOS expression was decreased by BG treatment dose-dependently in wt, PERK KO and XBP1 KO MEF cell lines while BG did not inhibit the CT-induced iNOS expression in IRE1α KO MEF cells, which is consistent with the NO production inhibition results above. In conclusion, these results suggest the antioxidant effect of BG by reducing iNOS expression followed by NO reduction, possibly via an IRE1α-dependent and XBP1-independent pathway.

### 3.3. Inhibitory Effect of BG on H_2_O_2_-Induced ROS Production

**Figure 3:**
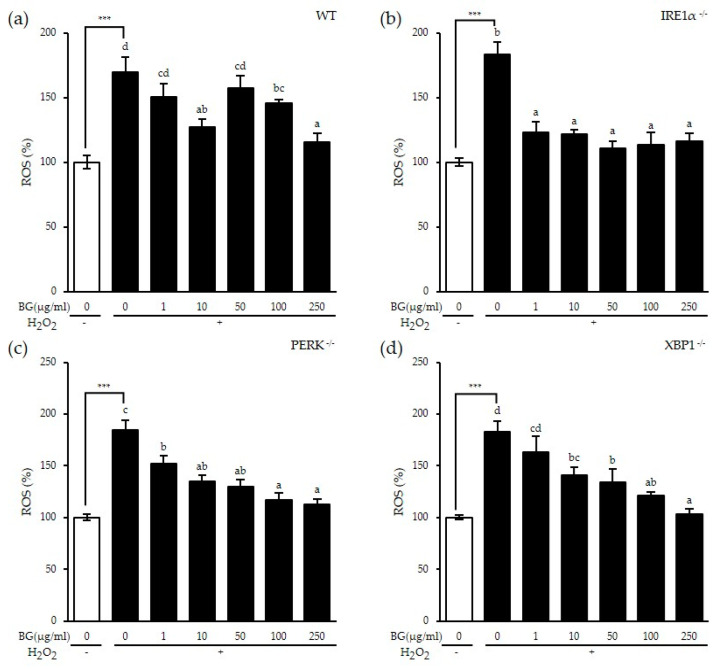
Inhibitory effect of BG on H_2_O_2_-induced reactive oxygen species (ROS) production in an IRE1α-dependent way. The (**a**) wt, (**b**) IRE1α KO, (**c**) PERK KO and (**d**) XBP1 KO MEF cell lines were pretreated with various concentrations of BG for 24 h followed by H_2_O_2_ stimulation for an additional 1 h. ROS level was determined using 2′, 7′-dichlorofluorescein diacetate (DCFH-DA) analysis. Data are shown as the means ± SD of three independent experiments. Significant differences between different concentrations were analyzed using Duncan’s multiple range test with one-way ANOVA and values labeled with different letters are significantly different (*p* < 0.05). Student’s *t*-test was performed to assess the difference between the control group and the H_2_O_2_-treated group in the absence of BG. *** *p* < 0.001. White bar represents negative control. BG, black ginseng.

Since we observed an inhibitory effect of BG on NO production, we investigated the cause of oxidation, ROS. Cells were pretreated with BG followed by H_2_O_2_ stimulation to induce ROS production. ROS levels increased significantly with H_2_O_2_ treatment in all cell lines as expected (*p* < 0.001). In wt MEF, PERK KO MEF cells and XBP1 KO MEF cells, H_2_O_2_-induced ROS was significantly decreased with BG treatment in a dose-dependent way (Figure 3a,c,d). However, BG completely inhibited H_2_O_2_-induced ROS production in IRE1α KO MEF cells, suggesting that BG inhibits ROS production through the IRE1α pathway (Figure 3b). Overall, our in vitro results indicate an antioxidant effect of BG on NO production by inhibiting ROS and iNOS production in an IRE1α-dependent and XBP1-independent pathway.

### 3.4. Cytotoxic and Inhibitory Effects of BG on ROS Production in Zebrafish Embryos

Since we observed the antioxidant function of BG in vitro, we investigated whether ROS levels can be inhibited by BG in vivo.

Zebrafish are known to share about 70–80% homogeneous sequences and similar functional roles with mammals, including humans [19] In addition, the zebrafish nervous system and various tissue structures are very similar to those of humans, making it an appropriate in vivo model for the study of various human diseases [20].

**Figure 4:**
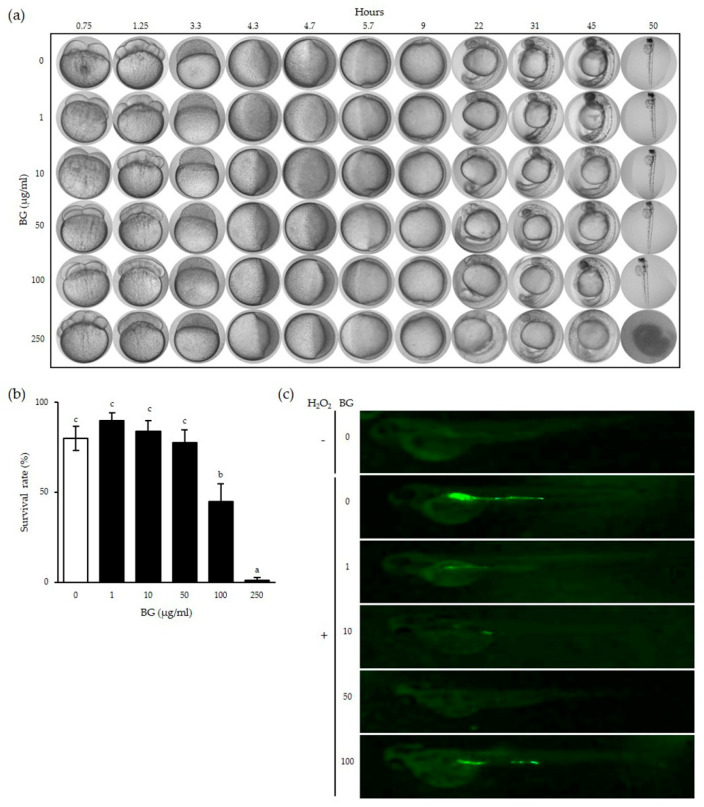
Cytotoxic and inhibitory effects of BG on ROS production in zebrafish embryos. Zebrafish eggs were incubated with BG in egg water for the indicated time (**a**). The survival rates of zebrafish eggs were determined by counting the hatched eggs (**b**). Zebrafish embryos were pretreated with the indicated concentration of BG for 24 h followed by additional 1 h H_2_O_2_ stimulation and the pictures were taken (**c**). Data are shown as the means ± SD of three independent experiments. Significant differences between different concentrations were analyzed using Duncan’s multiple range test with one-way ANOVA, and values labeled with different letters were significantly different (*p* < 0.05). White bar represents negative control. BG, black ginseng.

Zebrafish embryos were exposed to BG at different concentrations to observe the toxicity of BG at each indicated time (Figure 4a). No deterioration of embryo development or variation in hatching time was observed after exposure to BG at concentrations up to 100 µg/mL. However, embryos treated with 250 µg/mL BG showed slower development compared to that of the control embryos and failed to develop fully to hatching. The survival rates of embryos treated with above 100 µg/mL BG were significantly decreased (*p* < 0.001), indicating that a 50 µg/mL or lower BG concentration was not toxic in vivo (Figure 4b).

Next, the H_2_O_2_-induced changes in ROS levels were measured in zebrafish pretreated with BG for 24 h. H_2_O_2_-induced ROS is represented as the green fluorescent protein (GFP) shown in Figure 4c. Most of the GFP was located near the digestive system of the zebrafish. However, GFP imaging dimmed with pretreatment of BG at concentrations of 1–50 µg/mL in a dose-dependent manner, with no GFP shown with the 50 µg/mL BG pretreatment. However, GFP reappeared at the 100 µg/mL BG concentration, suggesting that a high concentration of BG may have a toxic effect already confirmed in Figure 4a,b. These results show that BG can inhibit ROS production up to 50 µg/mL in a dose-dependent manner in vivo.

### 3.5. Inhibitory Effect of BG on a NAFLD Mouse Model

In order to confirm the antioxidant effects of BG, the NAFLD mouse model was used in vivo. We chose an NAFLD mouse model to examine whether BG affects ER stress and inflammation, since NAFLD is a chronic disease associated with inflammation. It has been reported that NAFLD-induced mice exhibited liver inflammation, and BG reduced this phenotype by enhancing the antioxidant enzymes of the liver [21,22,23].

Initially, ICR male mice were fed a high-fat diet with 10% fructose in drinking water to induce inflammation; subsequently, the diet was supplemented with BG for eight weeks. Dietary intake, mice weights and food efficiency ratio were not changed by BG during the experiment [21].

**Figure 5:**
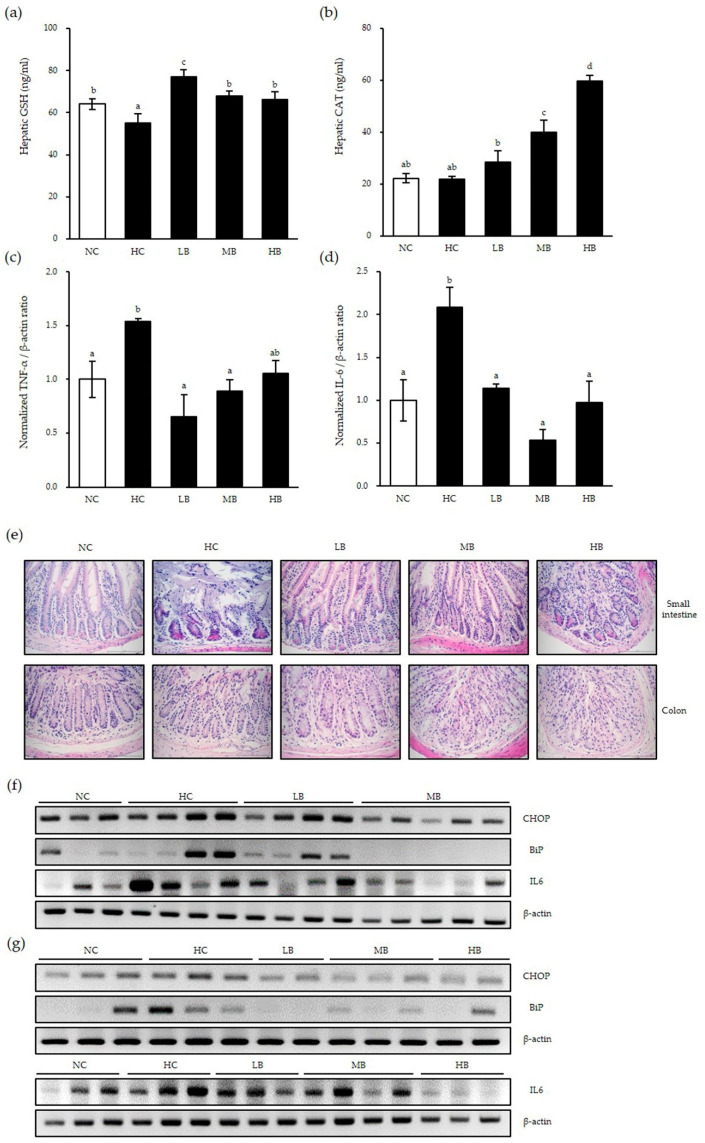
Antioxidant and anti-inflammatory effects of BG in nonalcoholic fatty liver disease (NAFLD)-induced mice. Liver tissue was harvested and examined using ELISA (**a,b**) and real-time qPCR (**c**,**d**). The small intestine and colon were harvested and stained with hematoxylin and eosin (H&E) (**e**). Whole tissues of small intestine (**f**) and colon (**g**) were harvested and total RNA was extracted for RT-PCR. β-actin was used as the loading control. Data are shown as the means ± SD of three independent experiments. Significant differences between different concentrations were analyzed using Duncan’s multiple range test with one-way ANOVA and values labeled with different letters are significantly different (*p* < 0.05). GSH, glutathione; CAT, catalase; NC, normal untreated control; HC, high-fat/high-fructose diet; LB, HC + 0.5% black ginseng; MB, HC + 1% black ginseng; HB, HC + 2% black ginseng. The mRNA expression level was normalized to β-actin and expressed relative to the NC control group, which was set to 1.

First, we examined whether there were antioxidant activities by BG in the liver of mice using ELISA assays. GSH protein expressions were decreased significantly in the HC group compared to that of the control diet group, as expected (Figure 5a). Low dose BG supplementation rescued the GSH expression significantly; in addition, medium and high-dose BG supplementation rescued the GSH expression level to that of the control group. Although CAT protein expression was not changed by the HC, BG supplementation significantly increased CAT expression in a dose-dependent manner, suggesting that BG enhances antioxidant enzyme activities in the liver of NAFLD mice (Figure 5b).

In order to determine whether BG affects the inflammatory response in the liver, expression levels of proinflammatory factors TNF-α and IL-6 mRNA were examined (Figure 5c,d). Compared with the control diet group, the HC group had significantly increased proinflammatory mRNA expressions in the liver, implying that NAFLD induces significant inflammation in the liver as expected. However, BG diet supplementation significantly decreased high-fat diet-induced TNF-α and IL-6 levels to a similar level as the NC group, regardless of the BG concentration. These results show that BG reduces inflammation by downregulating proinflammatory cytokines in the presence of inflammation.

Next, we examined inflammation and ER stress in the small intestine and colon of NAFLD mice, which are the first barriers for foreign substances. The morphology and inflammatory cell infiltration into the small intestine and colon were examined by H&E staining (Figure 5e). Compared to the control diet, the HC group showed significant inflammatory cell infiltration and shortened and destroyed villi in the small intestine. This pattern was abolished in the LB and MB groups, but not in the HB group, implying that high-dose BG supplementation might reverse the beneficial immunological effect of BG. In addition, all three BG-supplemented groups showed decreased inflammatory cell infiltration in the colon. Interestingly, goblet cell numbers were increased in the LB group compared to the control group.

Next, the expressions of ER stress markers and inflammatory cytokines were examined. Compared with the control diet, the HC group had increased expressions of CHOP, BiP and IL-6 mRNA in the small intestines (Figure 5f) and the colon (Figure 5g). Expression levels of CHOP, BiP and IL-6 were decreased in the small intestine of the LB and MB groups. Similarly, in the colon, all three BG-supplemented groups had markedly decreased CHOP, BiP and IL-6 expression levels from those of the control group. These results are consistent with our data in the liver above. In conclusion, BG supplementation reduced ER stress (as represented by BiP expression), thus preventing apoptosis (as represented by apoptosis-inducing ER stress marker CHOP expression) and, ultimately, reducing inflammation (as represented by IL-6 expression) in the liver and the intestines under inflammatory conditions such as those in NAFLD mice.

## 4. Discussion

Previous studies have shown that steaming ginseng nine times improves its preservative properties and efficacy [15]. This method resulted in increased radical scavenging activity due to the release of phenolic compounds and Maillard reaction products during the heat treatment process. This process makes BG contain relatively lower polar ginsenosides compared to the white and red ginseng, and 19 ginsenosides such as Rb1, Rb2, Rc, Rd, Re, Rf, Rg1, Rg6, F4, Rk3, Rh4, 20(S)-, 20(R)-Rg3, 20(S)-, 20(R)-Rs3, Rk1, Rg5, Rs4 and Rs5 were discovered. In addition, our previous study confirmed and identified 22 different types of ginsenosides of BG and their amounts such as Rg1, Re, Rf, Rh1 (S and R), Rh2 (S and R), Rb1, Rc, F1, Rb2, Rb3, F2, Rg3 (S and R), PPT (S and R), K, Rh2 (S and R), PPD and total ginsenosides in the leaf (22.94 mg/g) and roots (21.00 mg/g) [17,18].

Antioxidant and anti-inflammatory effects of BG have been demonstrated in many studies [24,25,26]. With these bioactive components, we showed reduction of lipid accumulation in the liver. However, there are few reports on the association of ER stress with the antioxidant and anti-inflammatory effects of BG. Herein, we used MEF cells knocked out of the ER pathway to determine how these functions are regulated under ER stress.

In this study, we showed that cytokine-induced ROS production and NO production were reduced significantly by BG treatment

Our data showed that H_2_O_2_-induced ROS production decreased with BG treatment in zebrafish. However, we believe that the reason the ROS expression level returned to its original state when using a BG concentration of 100 μg/mL is because a concentration of 100 μg/mL might be toxic to zebrafish.

In this study, we observed that BG enhanced the activity levels of antioxidant enzymes in the liver of NAFLD mice. We also observed that the expression levels of the ER stress factor BiP, the apoptosis factor CHOP, and the inflammatory factor IL-6 in the small and large intestines were higher in the HC group than in the NC group, and BG treatment of NAFLD mice reduced inflammation and ER stress significantly. Histological analysis of intestinal tissues showed that HFD-induced lymphocyte infiltration, an indicator of inflammation, was reduced by BG supplementation.

## 5. Conclusions

In conclusion, our study showed that BG inhibits ROS production followed by the regulation of ER stress, possibly in IRE1α-dependent and XBP1-independent ways, which results in the inhibition of inflammation.

Overall, our results show that BG supplementation can enhance both liver and intestinal health by enhancing the activity levels of antioxidant enzymes in the liver and reducing inflammation in the liver and the intestine via the IRE1α-dependent ER stress pathway, indicating that BG may be useful as a therapeutic option for application in inflammation-related diseases.

## Figures and Tables

**Table 1:** Experimental design of the study.

Experimental Group	Diet	Number of Mice
NC	Normal drinking water	10
HC	45% high-fat diet with 10% fructose in the drinking water	9
LB	45% high-fat diet supplemented with 0.5% BG with 10% fructose in the drinking water	9
MB	45% high-fat diet supplemented with 1% BG with 10% fructose in the drinking water	9
HB	45% high-fat diet supplemented with 2% BG with 10% fructose in the drinking water	9
Total		46

**Table 2:** Mouse primer sequences used in reverse transcription PCR, real-time qPCR.

	Gene	Accession Number	Primer Sequence (5′–3′)	bp
RT-PCR	BiP	NM_001163434.1	Forward: CTG GGT ACA TTT GAT CTG ACT GG Reverse: GCA TCC TGG TGG CTT TCC AGC CAT TC	398
	CHOP	NM_007837.4	Forward: CAC ATC CCA AAG CCC TCG CTC TC Reverse: TCA TGC TTG GTG CAG GCT GAC CAT	286
	IL-6	NM_001314054.1	Forward: CCG GAG AGG AGA CTT CAC AG Reverse: GGA AAT TGG GGT AGG AAG GA	421
	β-actin	NM_007393.5	Forward: TCT CCA GCA ACG AGG AGA AT Reverse: TGT GAT CTG AAA CCT GCT GC	348
Real-time qPCR	TNF-α	NM_013693.3	Forward: TGT CCC TTT CAC TCA CTG GC Reverse: CAT CTT TTG GGG GAG TGC CT	
	IL-6	NM_001314054.1	Forward: GGG ACT GAT GCT GGT GAC AA Reverse: TCC ACG ATT TCC CAG AGA ACA	
	β-actin	NM_007393.5	Forward: GACGTTGACATCCGTAAAG Reverse: CAGTAACAGTCCGCCT	

RT-PCR, reverse transcription polymerase chain reaction; BiP, binding immunoglobulin protein; CHOP, CCAAT/enhancer-binding homologous protein; IL-6, interleukin-6; real-time qPCR, real-time quantitative polymerase chain reaction; TNF-α, tumor necrosis factor-α; NF-κB, nuclear factor kappa light chain enhancer of activated B cells.

## Data Availability

Data is contained within the article.
